# Chicago Society of Physicians and Surgeons

**Published:** 1874-03

**Authors:** Plym. S. Hayes


***Chicago Society of Physicians and Surgeons.*** *Transactions at Meeting of January 26th*, 1874. *Reported by* PLYM. S. HAYES, M.D.

The Society met as usual in the parlor of the Grand Pacific Hotel, the President, Dr. Fisher, in the chair.

Drs. E. Andrews and W. Blanchard were elected to membership.

The Secretary read a paper written by the President, Dr. Fisher, on the Progress of Medicine. The paper reviewed the advances made in medical science to the present time, and compared the slow progress of this science before the commencement of the nineteenth century with its rapid advance since that time.

Dr. C. P. Simon reported the case of a young man who had taken about dr. iij. of tr. opium. When discovered, three hours before the doctor was summoned, the body was motionless and pulseless. When the doctor arrived he found the extremities cyanosed and cold; the neck and lower jaw rigid, the pupils dilated, and the iris brilliant and phosphorescent. No pulse could be detected, and there were no respirations. Upon applying his ear to the chest, the doctor thought he was able to detect feeble pulsations, which averaged thirty to the minute. Ten minutes after his arrival the pulsations had entirely ceased.

Dr. Etheridge then related the following case of opium poisoning. When he first saw the patient, there were twelve stertorous respirations and forty-eight pulsations to the minute. After a time the respirations ceased entirely for about eighty seconds, after which they were resumed with an audible sound, and then the respirations which were quiet and normal, gradually grew more and more shallow and stertorous until they again ceased. The periods of cessation varied from fifty to one hundred seconds; while those of respiration continued to take place from eight to ten minutes. During the cessation there was a tremor of the intercostal muscles. The restorative means used were, the stomach pump, and atropia, given by the mouth. Half an hour after the atropia had been given, the pupils gradually dilated until death occurred, eight hours after the doctor had been called.

Dr. Trimble cited two similar cases in which atropia and artificial respiration had been used; in one of the cases faradization had also been employed; both recovered.

Dr. Wilder related the case of a man who took tr. opium, oz. ij. Although emetics, belladonna, artificial respiration and friction were used, the patient died. The pupils dilated after the belladonna had been given.

Dr. Bartlett mentioned a case of poisoning from a belladonna plaster which had been applied for threatened mammary abscess. The patient was taking morphine all of the time. He also gave the case of a child who had taken tr. opium, dr. j. He was called soon after the drug had been taken, and not having a stomach pump at hand, introduced a catheter in place of the tube of the pump, filled the stomach with water and then reversed the child, when the contents of the stomach escaped.

Dr. Etheridge stated that larger doses of opium and belladonna could be borne when given together than when given separately; the toxic effect of the one neutralizing that of the other; and cited Dr. Brown-Sequard as authority.

Dr. Powell remarked, that he used after an operation one-half gr. of morphia and one-sixtieth gr. of atropia hypodermically; the action of the morphia being thus continued much longer than when given alone.

Dr. Trimble introduced a resolution requiring the President to appoint a committee of three on necrology at each annual meeting.

Dr. Danforth is expected to read at the next meeting, a paper on the pathology of the late cholera epidemic, illustrated by means of a solar microscope.

The society then adjourned.

---

*Meeting of Feb.* 9, 1874.

The Society met as usual in the parlor of the Grand Pacific Hotel; the President, Dr. Fisher, in the chair. Drs. J. H. Hollister and W. T. Montgomery were unanimously elected to membership.

Dr. Hyde read the following communication:

CHICAGO, Ill., Feb. 9, 1874.

*Dear Sir*—I have the pleasure of presenting to the Chicago Society of Physicians and Surgeons, in behalf of Assistant Surgeon John S. Billings, U. S. A., the librarian, the accompanying two volumes of the catalogue of the library of the Surgeon General's Office, U. S. Army.

Very respectfully, W. C. SPENCER,
*Surgeon U. S. Army.*

Dr. J. N. HYDE, Sec. Chicago Society of Physicians and Surgeons.

A vote of thanks was tendered to the Surgeon General.

Dr. Danforth's report on the Pathology of Endemic Cholera, was read by Dr. Bridge. The report mainly consisted of the history, necroscopy, and microscopical examination of two cases of patients that died in the cholera hospital last summer. Dr. Danforth explained the sections of normal and pathological intestines, which he had prepared. These were projected on the screen by means of a solar microscope. The instrument used was one of Browning's spectroscopic lanterns, with microscopic attachment. The lantern had been recently presented to Rush Medical College by Mr. A. C. Thomas, and kindly loaned to the Society by the college.

A vote of thanks was given to Dr. Danforth for the paper and exhibition of microscopical illustrations.

The following resolution was adopted:

*Resolved*, That the report be given to the Secretary for publication in some medical journal.

As the hour was late, the discussion of the paper was made the business of the next meeting.

The meeting then adjourned.

☞ Reports of meetings of the Central Illinois Medical Society, which have been placed in type, have been unavoidably crowded out of this No., but will appear next month.—EDS. JOURNAL.

